# Glutamate receptor-interacting protein 1 in D1- and D2-dopamine receptor-expressing medium spiny neurons differentially regulates cocaine acquisition, reinstatement, and associated spine plasticity

**DOI:** 10.3389/fncel.2022.979078

**Published:** 2022-11-03

**Authors:** He Chen, Limei Chen, Zhirong Yuan, Jiajie Yuan, Yitong Li, Yuesi Xu, Jieyi Wu, Lu Zhang, Guohua Wang, Juan Li

**Affiliations:** ^1^Department of Histology and Embryology, School of Basic Medical Sciences, Southern Medical University, Guangzhou, China; ^2^Key Laboratory of Functional Proteomics of Guangdong Province, Key Laboratory of Mental Health of the Ministry of Education, School of Basic Medical Sciences, Pediatric Center of Zhujiang Hospital, Southern Medical University, Guangzhou, China; ^3^School of Food and Biotechnology, Guangdong Industry Polytechnic, Guangzhou, China

**Keywords:** cocaine acquisition and reinstatement, GRIP1, D1-MSNs, D2-MSNs, spine plasticity

## Abstract

**Background:**

The nucleus accumbens (NAc) is involved in the expression of cocaine addictive phenotypes, including acquisition, extinction, and reinstatement. In the NAc, D1-medium spiny neurons (MSNs) encode cocaine reward, whereas D2-MSNs encode aversive responses in drug addiction. Glutamate receptor-interacting protein 1 (GRIP1) is known to be associated with cocaine addiction, but the role of GRIP1 in D1-MSNs and D2-MSNs of the NAc in cocaine acquisition and reinstatement remains unknown.

**Methods:**

A conditioned place preference apparatus was used to establish cocaine acquisition, extinction, and reinstatement in mouse models. GRIP1 expression was evaluated using Western blotting. Furthermore, GRIP1-siRNA and GRIP1 overexpression lentivirus were used to interfere with GRIP1 in the NAc. After the behavioral test, green fluorescent protein immunostaining of brain slices was used to detect spine density.

**Results:**

GRIP1 expression decreased during cocaine acquisition and reinstatement. GRIP1-siRNA enhanced cocaine-induced CPP behavior in acquisition and reinstatement and regulated associated spine plasticity. Importantly, the decreased GRIP1 expression that mediated cocaine acquisition and reinstatement was mainly driven by the interference of the GRIP1-GluA2 interaction in D1-MSNs and could be blocked by the interference of the GRIP1-GluA2 interaction in D2-MSNs. Interference with the GRIP1-GluA2 interaction in D1- and D2-MSNs decreased spine density in D1- and D2-MSNs, respectively.

**Conclusion:**

GRIP1 in D1- and D2-MSNs of the NAc differentially modulates cocaine acquisition and reinstatement. GRIP1 downregulation in D1-MSNs has a positive effect on cocaine acquisition and reinstatement, while GRIP1 downregulation in D2-MSNs has a negative effect. Additionally, GRIP1 downregulation in D1-MSNs plays a leading role in cocaine acquisition and reinstatement.

## Introduction

The characteristics of drug addiction including cocaine addiction are compulsive drug seeking and episodes of relapse despite prolonged periods of abstinence. During drug seeking, craving, and relapse, the nucleus accumbens (NAc) is one of the most important brain areas and is directly involved in the expression of addictive phenotypes (Quintero, 2013; Wang et al., 2016; Cannella et al., 2018; Lax and Sapozhnikov, 2019). Studies have shown that cocaine-induced dendritic spine plasticity of medium spiny neurons (MSNs) and glutamatergic neurotransmission in the NAc contribute to addictive behavior (Huang et al., 2009; Kim et al., 2011; Lüscher and Malenka, 2011; Quintero, 2013; Liu et al., 2018; Benneyworth et al., 2019).

The MSNs in the NAc are mainly segregated into two subtypes, those expressing dopamine receptor D1 (D1-MSNs) or dopamine receptor D2 (D2-MSNs). Normally, D1-MSNs encode cocaine reward, whereas D2-MSNs encode aversive responses in drug addiction (Hikida et al., 2010; Lobo et al., 2010; Kravitz et al., 2012; Soares-Cunha et al., 2020). The same molecules in different subtypes of MSNs can play similar or different regulatory roles in cocaine addiction (Chandra et al., 2015; Ceglia et al., 2017). Wiskott–Aldrich syndrome protein (WASP) family verprolin homologous protein 1 (WAVE1) regulates cocaine-associated behavioral actions in D1-MSNs, but not in D2-MSNs (Ceglia et al., 2017), while transcription factor early growth response 3 (Egr3) in D1-MSNs and D2-MSNs plays opposing roles in cocaine action (Chandra et al., 2015). Therefore, it is necessary to distinguish different cell subtypes when studying the regulatory role of the same molecule in disease.

Glutamatergic neurotransmission is predominantly dependent on the α-amino-3-hydroxy-5-methyl-4-isoxazolepropionic acid receptor (AMPAR) subtype. AMPARs are tetrameric complexes composed of four subunits, GluA1–4 (Gan et al., 2015). Among these, the GluA2 subunit is intriguing because of its action on Ca^2+^ trafficking. GluA2-containing AMPARs are impermeable to Ca^2+^, while GluA2-lacking AMPARs are Ca^2+^ permeable (Geiger et al., 1995; Cull-Candy et al., 2006). AMPARs lack newly formed spines induced by cocaine acquisition, which generates silent synapses and mediates cocaine addictive behavior (Huang et al., 2009; Terrier et al., 2016). During cocaine reinstatement, GluA2-lacking AMPARs increase in the postsynaptic membrane, which promotes cocaine-seeking behavior (Quintero, 2013; White et al., 2016).

Synaptic trafficking of AMPA receptors is tightly regulated by neural scaffolding proteins such as glutamate receptor interacting proteins 1 and 2 (GRIP1/2) (Dong et al., 1997, 1999; Takamiya et al., 2008) and proteins interacting with C kinase 1 (Lu and Ziff, 2005). GRIP1/2 usually have seven PSD-95/SAP90/DLG/ZO-1 (PDZ) domains (Dong et al., 1997, 1999); among these, PDZ4–6 domains can bind to the C-terminal domains of GluA2/3 subunits of AMPARs, which mediate GluA2/3 trafficking and thus affect synaptic organization and transmission (Dong et al., 1997). In cocaine addiction, GRIP1 knockout in the NAc enhances vulnerability to cocaine relapse (Briand et al., 2014), and calpain in the NAc core mediates the reconsolidation of drug reward memory through GRIP1 expression (Liang et al., 2017). Meanwhile, conditional deletion of GRIP1 in the medial prefrontal cortex (PFC) increases the motivation for cocaine and potentiates cue-induced reinstatement of cocaine seeking (Wickens et al., 2019). However, the role of GRIP1 in D1-MSNs and D2-MSNs of the NAc in cocaine acquisition and reinstatement remains unknown.

The present study aimed to pinpoint the role of GRIP1-GluA2 in D1-MSNs and D2-MSNs of the NAc in cocaine acquisition and reinstatement and associated spine plasticity.

## Materials and methods

### Mice

Male C57BL/6J mice were purchased from the Southern Medical University Animal Center (Guangzhou, China). All mice were 6–8 weeks old at the beginning of the treatment. Five mice were housed per cage under standard conditions with *ad libitum* access to rodent chow and water and a 12:12 h light/dark cycle (lights on at 8:00 a.m.). All behavioral tests were conducted during the light period.

All experimental procedures complied with the National Institutes of Health guidelines and were approved by the Institutional Animal Care and Use Committee of Southern Medical University.

### Viral constructs

GRIP1-overexpression and GRIP1-siRNA lentiviruses were packaged and supplied by Vector Builder at a titer of 2 × 10^8^ viral genomes per mL (v.g./mL). For GRIP1 overexpression, full-length GRIP1 cDNA (NM_001358810.1) was amplified from a mouse brain cDNA library and cloned into a human synapsin I (hSyn) promoter lentivirus vector tagged with eGFP. For GRIP1-siRNA, the sequence “5′-rArGrA rUrArA rCrUrC rArGrA rCrGrA rGrCrA rArGrA rGrAG T-3′ 5′-rArCrU rCrUrC rUrUrG rCrUrC rGrUrC rUrGrA rGrUrU rArUrC rUrUrC-3′,” which has been reported to successfully inhibit the expression of GRIP1 in mouse cells (Luo et al., 2012), was cloned into an hSyn promoter lentivirus vector tagged with eGFP.

PSD-95/SAP90/DLG/ZO-1 (PDZ) domain 4/5 and GluA2-dn lentiviruses were packaged and supplied by OBiO Technology (Shanghai) Corp., Ltd. at a titer of 2 × 10^8^ v.g. per mL. For hSyn-DIO-GRIP1-PDZ4/5-eGFP, nucleotides coding for aa441–655 (PDZ4-5) of GRIP1 were amplified from a rat brain cDNA library, which successfully inhibits the interaction of GRIP1 and GluA2 by interfering with the GluA2 binding site of GRIP1 in mouse hippocampal neurons (Heisler et al., 2014). For hSyn-DIO-GluA2-dn, nucleotides coding for aa853–883 of GluA2 were also amplified from a rat brain cDNA library, which inhibited the interaction of GluA2 and GRIP1 by interfering with the GRIP1 binding site of GluA2 in mouse hippocampal neurons (Heisler et al., 2014).

Adeno-associated viruses (AAV2/9-Drd1-Cre-mCherry, AAV2/9-Drd2-Cre-mCherry) were packaged and supplied by BrainVTA (Wuhan) Co., Ltd. at a titer of 4 × 10^12^ v.g. per mL. These promoters can initiate the expression of target proteins specifically in D1-MSNs or D2-MSNs of the NAc, as reported by previous research (Ying et al., 2022).

The final concentrations in the lentivirus and AAV cocktail for injections were 1.5 × 10^8^ v.g./mL (Lentivirus) or 1 × 10^12^ v.g./mL (AAV).

### Stereotactic surgeries and viral injections

For all surgical procedures, mice were anesthetized with 1.5% isoflurane at an oxygen flow rate of 1 L/min and positioned in a stereotaxic frame (Stoelting Instruments) on a warm pad. Eyes were lubricated with an ophthalmic ointment. The fur was shaved, and the incision site was sterilized with betadine/ethanol swabs before surgery. Subcutaneous carprofen (5 mg/kg) was provided perioperatively and for three days postoperatively for analgesia. For lentivirus or lentivirus and AAV cocktail injections into the NAc, 0.4 μL of the virus cocktail was bilaterally injected with a 5 μL Hamilton syringe with a 33-gauge flat needle at a constant speed of 40 nL/min. Stereotaxic coordinates for the NAc are (from Bregma) + 1.4 mm, lateral ± 0.65 mm, dorsal/ventral - 4.75 mm. After each injection, the needle was left at the injection site inside the brain for an additional 5 min to aid diffusion from the needle tip and to prevent backflow. The needle was then slowly retracted, and the scalp incision was closed with stylolite. Before the start of the behavioral tests, mice were housed 3 weeks postoperatively.

### Conditioned place preference procedures

For cocaine acquisition training, the conditioned place preference (CPP) procedure was performed as follows: the CPP apparatus consisted of two conditioning compartments and a central connecting compartment. The two conditioning compartments had distinct wall patterns, floor textures, and lighting to allow mice to distinguish between them. The conditioning side was the cocaine-paired chamber, and the non-conditioning side was the saline-paired chamber. On Day 1, naive mice had free access to the entire apparatus for a 20-min session to reduce any effects of the experimental environment by adapting mice to the procedure. Mice that showed a strong unconditional preference for either side chamber (i.e., >780 s) were excluded from the experiments. On Day 2, baseline preference was assessed by placing the mice in the center chamber of the CPP apparatus and allowing them to explore all three chambers freely for 20 min. On the following day, the mice were trained for 8 consecutive days with alternating injections of cocaine (20 mg/kg, i.p., Qinghai Pharmaceutical Factory Co., Ltd., Qinghai, China) or an equivalent volume of saline and confined to a conditioning chamber for 20 min after each injection before being returned to their home cages. On Day 11, the final preference test was identical to the initial baseline preference assessment. The CPP score was defined as the time (in seconds) spent in the cocaine-paired chamber minus the time spent in the saline-paired chamber during the CPP tests.

For cocaine reinstatement training, the three-box biased CPP apparatus as described above and a two-box unbiased CPP apparatus, which had two 25 cm × 25 cm boxes with black and white stripes or squares separately, were used. The CPP procedure was performed as follows: On Day 1, baseline preference was assessed by placing the mice in the central chamber of the CPP apparatus and allowing them to explore all chambers freely for 20 min. On the following day, the mice were trained for 6 consecutive days with alternating injections of cocaine (10 mg/kg, i.p.) or an equivalent volume of saline and confined to a conditioning chamber for 20 min after each injection before being returned to their home cages. On Day 8, the cocaine preference test was identical to the initial baseline preference assessment. Furthermore, all mice received extinction training and were allowed to explore all chambers freely for 20 min each day without any drug injections, and the behavior performance was monitored until the entire group of addicted mice recovered. Reinstatement was induced by a priming injection of cocaine (10 mg/kg) before freely exploring all chambers for 20 min.

### Tissue sample preparation

After the preference test session, animals were decapitated at several time points. The NAc tissues were dissected using a brain blocker and homogenized in RIPA lysis buffer containing protease and phosphatase inhibitors for 30 min at 4°C. Subsequently, the homogenate was centrifuged at 14,000 rpm for 10 min and the supernatant was collected. All of the above procedures were performed at low temperature (0–4°C). Protein quantification was performed using the BCA Protein Assay Kit (Beyotime Biotechnology, Wuhan, China) and then normalized by diluting the samples with RIPA lysis buffer. The protein samples were mixed with 25% by volume of 5X SDS loading buffer before boiling for 5 min and stored at -80°C until the next step.

### Western blotting

GRIP1 expression in the NAc was measured by using Western blotting. Equal amounts of protein (40 μg) were loaded into each well of 8% Tris-glycine SDS-PAGE and electrophoretically transferred onto PVDF membranes. The membranes were blocked in 5% non-fat milk for 2 h at room temperature (RT) and incubated for 24 h at 4°C with mouse anti-GRIP1 (1:2,000; BD Biosciences, San Jose, CA, USA) and rabbit anti-β-actin (1:5,000, Bioss Antibodies, Beijing, China) primary antibody solutions. Following washing, the membranes were incubated with the secondary antibody, goat anti-mouse or anti-rabbit IgG conjugated with horseradish peroxidase (1:5,000, Bioss Antibodies, Beijing, China), for 1 h at RT. The blots were detected by super enhanced chemiluminescence and imaged on the Tanon Image system (Tanon Science & Technology Co., Ltd., Shanghai, China). All immunoblot analyses were performed in three or more replicates to obtain consistent results. Immunoblot signals were quantified using ImageJ (U.S. National Institutes of Health, Bethesda, MD, USA). GRIP1 levels were expressed as fold changes compared to the naive group.

### Immunohistochemistry

Mice were anesthetized with 10% chloral hydrate and subjected to transcardiac perfusion with 0.1 M phosphate-buffered saline (PBS) followed by 4% paraformaldehyde in 0.1 M PBS. Brains were removed and postfixed in 4% paraformaldehyde/PBS for an additional 12 h and then immersed in 20% sucrose/PBS for cryoprotection. After quick freezing, 25 μm thick coronal brain slices were sectioned using a cryostat (Leica CM, 1950, Wetzlar, Germany). For GFP expression in the lentiviral vector-injected animals, brain sections were examined using a fluorescence microscope. NAc-containing brain sections were washed with PBS and then immunostained with rabbit anti-GFP antibody (1:500, ab290, Abcam, Cambridge, USA) overnight at 4°C and Alexa Fluor 488-conjugated anti-rabbit secondary antibody (1:200, A-21206, Invitrogen, Carlsbad, CA, USA) for 1.5 h at RT.

### Confocal imaging and dendrite spine analysis

Fluorescent dendrite images of MSNs were captured using a Laser Scanning Confocal Microscope (Zeiss LSM 880, Germany). Individual neurons were visualized under a 63× oil immersion objective lens with 4× zoom at an optical slice thickness of 0.4 μm intervals along the z-axis. Individual MSNs in the NAc were chosen for spine analysis based on the following criteria (Lobo et al., 2010): (I) there was minimal or no overlap with other labeled cells to ensure that processes from different cells would not be confused; (II) at least three primary dendrites were visible for the cell; and (III) distal dendrites (terminal dendrites or close to the terminal dendrite) were present to be examined. For morphological analyses, spines were counted within 1–4 dendrite segments of ∼20 μm length on the third dendrites. For each group, 4–8 neurons per mouse were analyzed. All measurements were made with Microscopy Image Analysis Software (Imaris, 9.0.1, Switzerland).

Protrusions from dendrites were classified into four types based on their length and head diameter as follows (Lee et al., 2006): class 1, stubby spines with dendrite protrusions < 0.5 μm long and no discernible head; class 2, mushroom-shaped spines with dendrite protrusions with a head diameter >0.5 μm or >2× the spine neck diameter; class 3, thin spines with dendrite protrusions ranging from 0.5 to 3.0 μm in length and small head diameter <0.5 μm; class 4, filopodial extensions with filamentous protrusion longer than 3.0 μm.

### Statistical analyses

All statistical analyses were performed using GraphPad Prism 8.0 or SPSS 22.0 software. Behavioral analyses were performed using unpaired *t test*, two-way ANOVA, or one-way ANOVA followed by the Bonferroni *post-hoc* test. When the two-way ANOVA showed a significant interaction, simple main effects analyses were conducted separately. Student’s *t*-test was used to determine statistical significance between the two datasets. Data are presented as the means ± standard errors of the means (SEM). Significance was set at *P* < 0.05. The statistical significance is indicated by *P < 0.05, **P < 0.01, or ***P < 0.001.

## Results

### Glutamate receptor-interacting protein 1 in the nucleus accumbens regulates cocaine acquisition and associated spine plasticity

**FIGURE 1:**
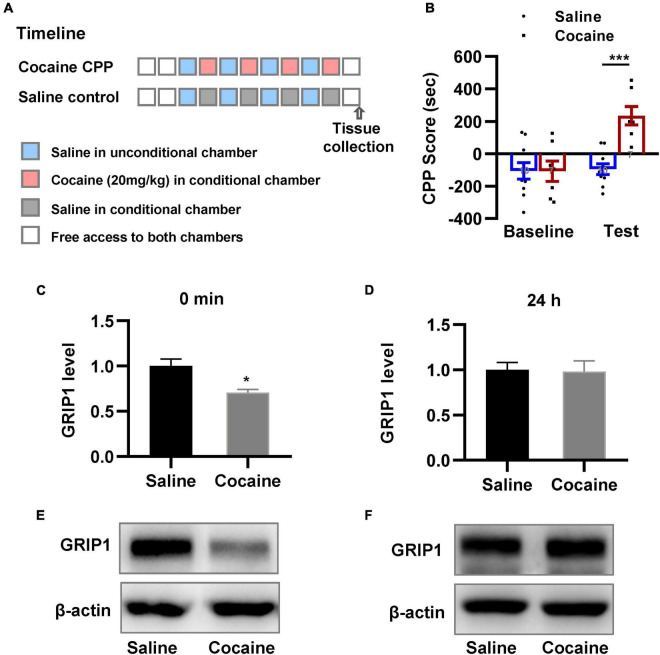
GRIP1 expression decreases after cocaine acquisition. **(A)** The timeline of cocaine or saline treatment applied for detecting GRIP1 expression in the NAc. **(B)** Cocaine-induced increased preference for the cocaine box. Unpaired *t* test. *n* = 8 mice per group. **(C–F)** The expression of GRIP1 in the NAc decreased at 0 min after cocaine acquisition compared to the saline control group **(C,E)** and returned to baseline at 24 h after cocaine acquisition **(D,F)**. The saline treatment group was set as 1 for quantification. Student’s *t*-test. *n* = 6 mice per group. NAc, nucleus accumbens. *P < 0.05, **P < 0.01, and ***P < 0.001.

We examined the changes in GRIP1 expression in the NAc. We trained mice using the CPP paradigm (Figure 1A); mice were divided into two groups, in which one received alternative saline or 20 mg/kg cocaine treatment, and another always received saline treatment as a control. The results showed that mice exhibited an increased preference for the cocaine chamber compared to the saline control after 8 days of CPP treatment (Figure 1B, unpaired *t test*, *t* = 6.729, *P* < 0.001). After the CPP test, NAc samples were acquired to detect GRIP1 expression at the 0 min and 24 h time points; we found that GRIP1 expression in the NAc decreased at 0 min compared to the saline control (Figures 1C,E, Student’s *t*-test, *t* = 3.450, *P* = 0.026) and returned to the original level at 24 h after the CPP test (Figures 1D,F, Student’s *t*-test, *t* = 0.142, *P* = 0.894). The results indicated that GRIP1 may be involved in cocaine acquisition.

**FIGURE 2:**
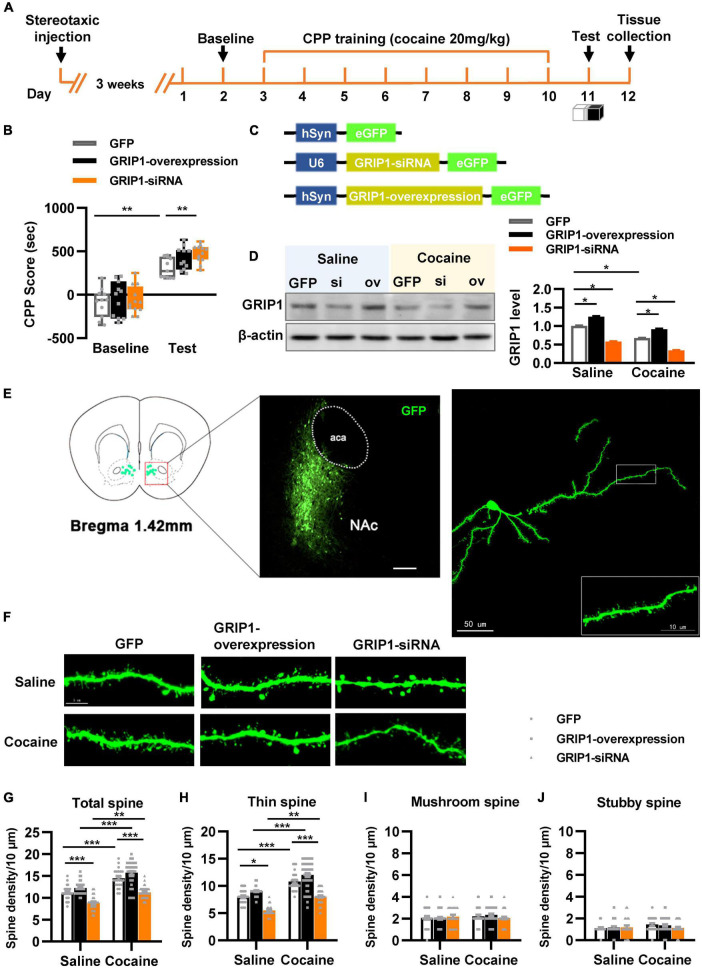
GRIP1 interference enhances cocaine acquisition and decreases spine density. **(A)** Experimental design for the behavioral tests and spine analysis. Three-compartment biased CPP models were used. **(B)** GRIP1-siRNA increased cocaine-induced place preference and GRIP1 overexpression did not affect cocaine-induced place preference. **(C)** Viral vectors used to interfere with the expression of GRIP1. **(D)** The expression of GRIP1 in the NAc interfered with GRIP1-siRNA and GRIP1 overexpression at 0 min after cocaine acquisition. **(E)** Representative images of the injection site (left) and spine densities on terminal dendrites (right). **(F)** Representative images of GFP-labeled dendrites. **(G–J)** The statistical data for total spine, thin spine, mushroom spine, and stubby spine density. *n* = 20–30 dendrites obtained from 8–10 mice per group. *P < 0.05, **P < 0.01, and ***P < 0.001.

To test our hypothesis, GRIP1-siRNA and GRIP1-overexpressing lentiviruses with the human synapsin I promoter tagged with eGFP (Figure 2C) were constructed and delivered into the NAc by stereotaxic injection, which successfully inhibited or overexpressed GRIP1 in the NAc after saline or cocaine treatment (Figure 2D, *P* < 0.05). After 3 weeks of recovery, the mice were subjected to the 3-compartment biased CPP paradigm (Figure 2A). The behavioral test results showed that GRIP1 inhibition by GRIP1-siRNA significantly enhanced cocaine-induced place preference (Figure 2B, two-way ANOVA followed by Bonferroni *post-hoc* test, *F*_virus(2,70)_ = 6.156, *P* = 0.006; *F*_drug (1,70)_ = 166.118, *P* < 0.001; *F*_interaction (2,70)_ = 1.262, *P* = 0.290), but GRIP1 overexpression did not affect cocaine-induced place preference (Figure 2B, *P* = 0.094, GRIP1 overexpression test vs. GFP test group). After the behavioral test, brain slices containing the NAc area around bregma 1.42 mm of the mice were used for GFP immunofluorescence detection, and the proximal terminal dendrites of MSNs were used to analyze spine plasticity (Figure 2E). We found that although GRIP1 interference by GRIP1-siRNA displayed a decreased total spine (Figures 2F,G, Two-way ANOVA, *F*_virus (2,172)_ = 74.016, *P* < 0.001; *F*_drug(1,172)_ = 66.555, *P* < 0.001; *F*_interaction(2,172)_ = 0.769, *P* = 0.731) and thin spine density (Figures 2F,H, Two-way ANOVA, *F*_virus(2,172)_ = 47.456, *P* < 0.001; *F*_drug(1,172)_ = 85.553, *P* < 0.001; *F*_interaction(2,172)_ = 4.924, *P* < 0.001) compared with GFP group after cocaine treatment, they also exhibit significant increase compared with their respective saline treatment group. Furthermore, it had no changes in mushroom spines (Figures 2F,I, Two-way ANOVA, *F*_virus(2,172)_ = 3.104, *P* = 0.047; *F*_drug(1,172)_ = 12.595, *P* < 0.001; *F*_interaction(2,172)_ = 2.295, *P* = 0.104) and stubby spines (Figures 2F,J, *F*_virus(2,172)_ = 7.273, *P* = 0.001; *F*_drug(1,172)_ = 11.794, *P* < 0.001; *F*_interaction(2,172)_ = 2.129, *P* = 0.122) density. Meanwhile, GRIP1 overexpression did not affect the cocaine-induced increase in dendritic spine density (*P* > 0.05). The results indicated that GRIP1 in the NAc regulated cocaine acquisition and basal spine density, but have no obvious effect on cocaine-induced increase in spine density.

### Glutamate receptor-interacting protein 1-GluA2 in D1-medium spiny neurons and D2-medium spiny neurons in the nucleus accumbens differentially regulates cocaine acquisition and associated spine plasticity

**FIGURE 3:**
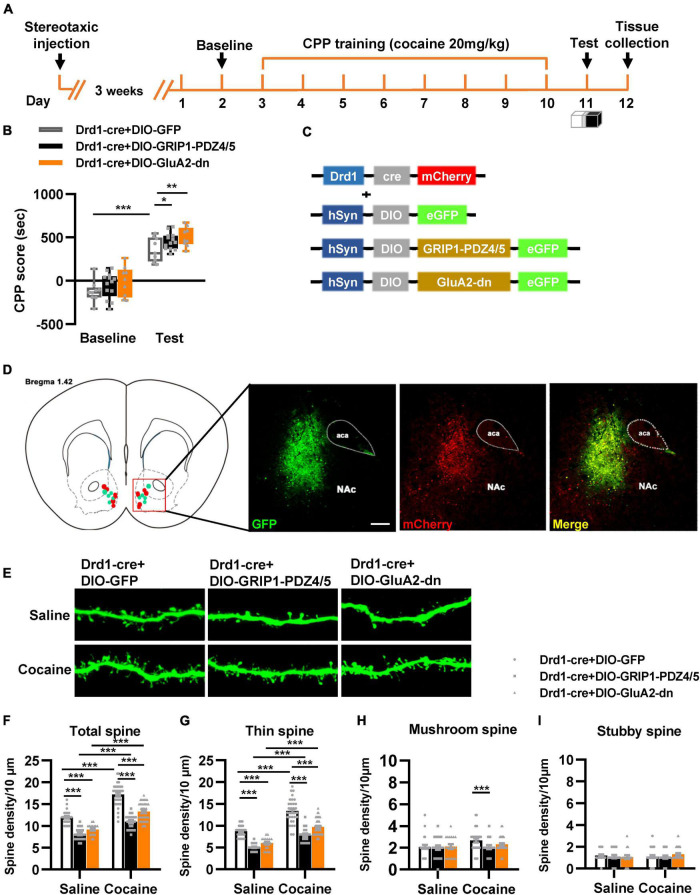
Interference with GRIP1 and GluA2 interaction in D1-MSNs enhances cocaine acquisition and decreases spine density of D1-MSNs. **(A)** Experimental design for the behavioral tests and spine analysis. Three-compartment biased CPP models were used. **(B)** GluA2-dn expressed in D1-MSNs increased cocaine-induced place preference; GRIP1-PDZ4/5 expressed in D1-MSNs did not affect cocaine-induced place preference. **(C)** Viral vectors used to interfere with the interaction of GRIP1 and GluA2 in D1-MSNs. **(D)** Representative images of the injection site. **(E)** Representative images of GFP-labeled dendrites. **(F–I)** The statistical data for total spine, thin spine, mushroom spine, and stubby spine density. *n* = 20–30 dendrites obtained from 8–10 mice per group. *P < 0.05, **P < 0.01, and ***P < 0.001.

Next, we evaluated the function of GRIP1 in D1-MSNs and D2-MSNs in regulating cocaine acquisition. We used Drd1-Cre adeno-associated virus (AAV) to initiate the overexpression of the GluA2 binding site of GRIP1 (GRIP1-PDZ4/5) or GluA2-dn (harboring the GRIP1 binding site of GluA2) in D1-MSNs (Heisler et al., 2014) (Figures 3C,D), which may interfere with the interaction between GRIP1 and GluA2 in D1-MSNs. Furthermore, the behavioral and spine analysis experiments were carried out following the procedure shown in Figure 3A. We found that disturbing the interaction of GRIP1 and GluA2 with GRIP1-PDZ4/5 and GluA2-dn in D1-MSNs enhanced cocaine-induced place preference (Figure 3B, two-way ANOVA, *F*_virus(2,58)_ = 6.867, *P* = 0.002; *F*_drug(1,58)_ = 198.626, *P* < 0.001; *F*_interaction(2,58)_ = 0.028, *P* = 0.972). Meanwhile, the spine analysis results showed that both GRIP1-PDZ4/5 and GluA2-dn displayed a decrease in total spines (Figures 3E,F, Two-way ANOVA followed by simple main effects analysis, *F*_virus(2,155)_ = 94.923, *P* < 0.001; *F*_drug(1,155)_ = 174.069,*P* < 0.001; *F*_interaction(2,155)_ = 4.903, *P* = 0.009) and thin spines (Figures 3E,G, Two-way ANOVA followed by simple main effects analysis, *F*_virus(2,155)_ = 87.047, *P* < 0.001; *F*_drug(1,155)_ = 156.938, *P* < 0.001; *F*_interaction(2,155)_ = 3.4048, *P* = 0.036) density of D1-MSNs compare with GFP group after cocaine treatment, but they also exhibit significant increase compared with their respective saline treatment group. Furthermore, it almost had no effect on mushroom spines (Figures 3E,H, Two-way ANOVA, *F*_virus(2,155)_ = 1.735, *P* = 0.180; *F*_drug(1,155)_ = 3.568, *P* = 0.061; *F*_interaction(2,186)_ = 1.204, *P* = 0.303) or stubby spines density (Figures 3E,I, Two-way ANOVA, *F*_virus(2,155)_ = 0.283, *P* = 0.754; *F*_drug(1,155)_ = 0.130, *P* = 0.046; *F*_interaction(2,155)_ = 0.975, *P* = 0.379) in D1-MSNs. Our data indicated that the interaction between GRIP1 and GluA2 regulated cocaine acquisition and basal spine density in D1-MSNs, but have no obvious effect on cocaine-induced increase in D1-MSNs spine density.

**FIGURE 4:**
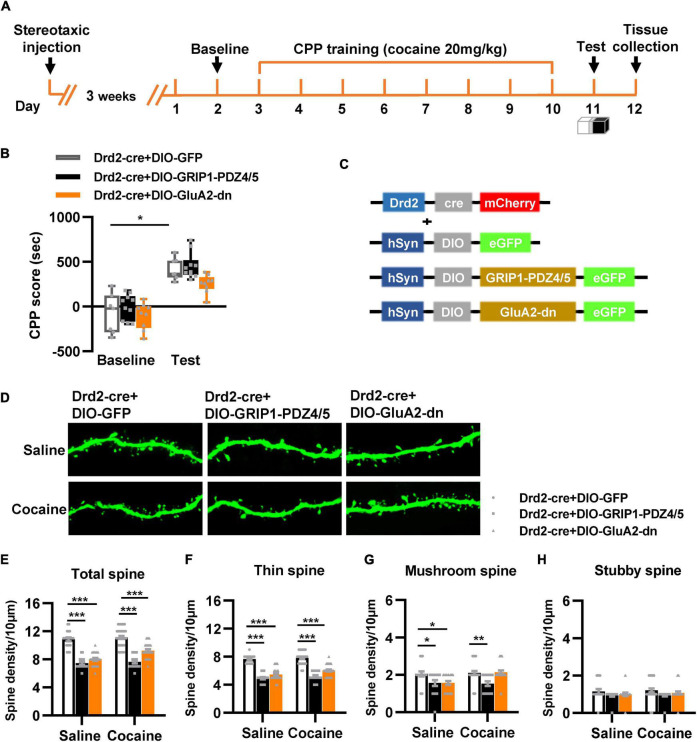
Interference with GRIP1 and GluA2 interaction in D2-MSNs blocks cocaine acquisition and decreases spine density of D2-MSNs. **(A)** Experimental design for the behavioral tests and spine analysis. Three-compartment biased CPP models were used. **(B)** GluA2-dn expressed in D2-MSNs blocked cocaine-induced place preference; GRIP1-PDZ4/5 expressed in D2-MSNs did not affect cocaine-induced place preference. **(C)** Viral vectors used to interfere with the interaction of GRIP1 and GluA2 in D1-MSNs. **(D)** Representative images of GFP-labeled dendrites. **(E–H)** The statistical data for total spine, thin spine, mushroom spine, and stubby spine density. *n* = 20–30 dendrites obtained from 8–10 mice per group. *P < 0.05, **P < 0.01, and ***P < 0.001.

Next, we used Drd2-Cre AAV to initiate GRIP1-PDZ4/5 or GluA2-dn in D2-MSNs following the procedure in Figures 4A,C. The behavioral test results showed that GRIP1-PDZ4/5 and GluA2-dn in D2-MSNs did not affect cocaine-induced place preference (Figure 4B, *F*_virus(2,56)_ = 0.401, *P* = 0.672; *F*_drug(1,56)_ = 101.101, *P* < 0.001; *F*_interaction(4,56)_ = 0.006, *P* = 0.994). Meanwhile, the spine analysis results showed that CPP training did not affect the dendritic spine density of D2-MSNs (Figures 4E–H, *P* > 0.05), but GRIP1-PDZ4/5 and GluA2-dn decreased the basal total spine density of D2-MSNs (Figures 4D,E, Two-way ANOVA, *F*_virus(2,137)_ = 24.071, *P* < 0.001; *F*_drug(1,137)_ = 3.388, *P* = 0.020; *F*_interaction(2,137)_ = 1.672, *P* = 0.128), and it was mainly driven by a decrease in basal thin spine density (Figures 4D,F, Two-way ANOVA, *F*_virus(2,137)_ = 129.720, *P* < 0.001; *F*_drug(1,137)_ = 5.435, *P* < 0.001; *F*_interaction(2,137)_ = 0.999, *P* = 0.371), and basal mushroom spine density (Figures 4D,G, Two-way ANOVA followed by simple main effects analysis, *F*_virus(2,137)_ = 8.969, *P* < 0.001; *F*_drug(1,137)_ = 4.016, *P* = 0.047; *F*_interaction(2,137)_ = 3.874, *P* = 0.023) of D2-MSNs, intriguingly, this kind of effect on basal spine density continued after cocaine treatment. Futhermore, GRIP1-PDZ4/5 and GluA2-dn did not affect stubby spine density of D2-MSNs (Figures 4D,H, Two-way ANOVA, *F*_virus(2,137)_ = 2.655, *P* = 0.074; *F*_drug(1,137)_ = 0.270, *P* = 0.604; *F*_interaction(2,137)_ = 0.050, *P* = 0.951). Our data indicate that the interaction between GRIP1 and GluA2 in D2-MSNs was also involved in cocaine acquisition and regulated basal spine density of D2-MSNs, what’s more, it had an opposite regulatory role to that in D1-MSNs.

### Glutamate receptor-interacting protein 1 in the nucleus accumbens regulates cocaine reinstatement and associated spine plasticity

**FIGURE 5:**
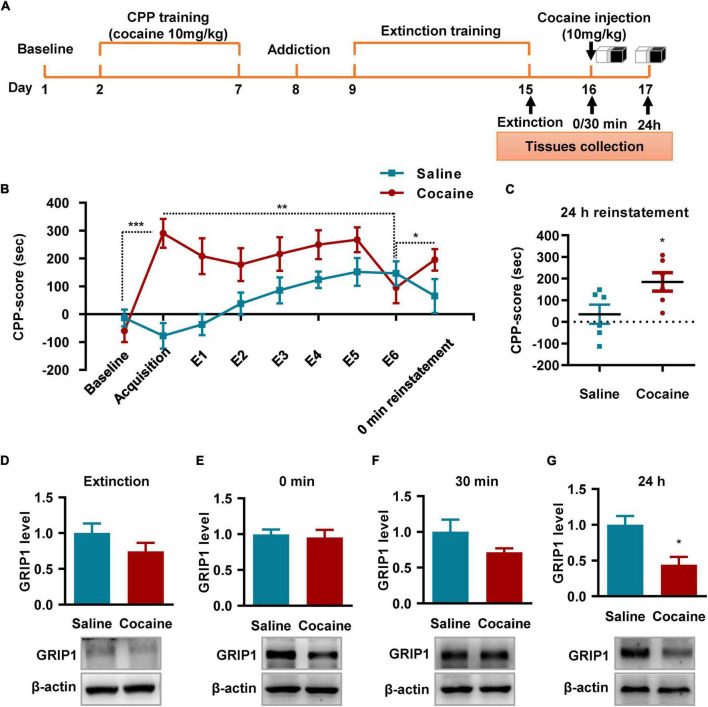
GRIP1 expression decreases at 24 h after cocaine-induced reinstatement. **(A)** The timeline of cocaine or saline treatment applied for detecting GRIP1 expression in the NAc. Three-compartment biased CPP models were used. **(B)** The CPP score during the test schedule. *n* = 10 mice per group. **(C)** The CPP score at 24 h after cocaine-induced reinstatement. Student’s *t*-test, *n* = 6 mice per group. **(D–G)** The expression of GRIP1 in the NAc decreased at 24 h after cocaine-induced reinstatement compared to the saline control group **(C,E)**, but there were no significant changes after extinction **(D)** or at 0 min **(E)** or 30 min **(F)** after cocaine-induced reinstatement compared to the saline control. The saline treatment group was set as 1 for quantification. Student’s *t*-test, *n* = 4 mice per group. *P < 0.05, **P < 0.01, and ***P < 0.001.

Next, we detected the role of GRIP1 in regulating cocaine reinstatement and associated spine plasticity. We induced cocaine reinstatement in a mouse model (Figure 5A). The behavioral test results showed that a 10 mg/kg cocaine dosage induced cocaine place preference after 6 days of alternating cocaine and saline injection (Figure 5B, Acquisition vs. Baseline, *t* = 5.282, *P* < 0.001); 6 days of extinction training resulted in loss of cocaine place preference (Figure 5B, E6 vs. acquisition, *t* = 2.604, *P* = 0.026). Furthermore, one injection of 10 mg/kg cocaine induced cocaine reinstatement and exhibited an increased CPP score compared to that of extinction Day 6 (Figure 5B, reinstatement vs. E6, *t* = 2.472, *P* = 0.033). According to the behavioral performance, high CPP score mice were chosen to detect GRIP1 expression (Figure 5A) at 0 min reinstatement; the remaining mice continued to be used for behavioral tests. The reinstated mice showed high CPP scores (Figure 5C, cocaine vs. saline, *t* = 2.427, *P* = 0.036) in the 24 h test, which was used to detect the expression of GRIP1 protein in NAc. The results showed that GRIP1 expression decreased at 24 h in reinstated mice after cocaine injection (Day 17, Figure 5G, *t* = 3.388, *P* = 0.012); there were no obvious changes at 0 min (Figure 5E, *t* = 0.356, *P* = 0.74) or 30 min (Figure 5F, *t* = 0.851, *P* = 0.123) after the CPP test on Day 16 or on extinction Day 6 (Figure 5D, *t* = 1.418, *P* = 0.229) compared to the saline control. The data indicate that GRIP1 in the NAc may regulate cocaine reinstatement.

**FIGURE 6:**
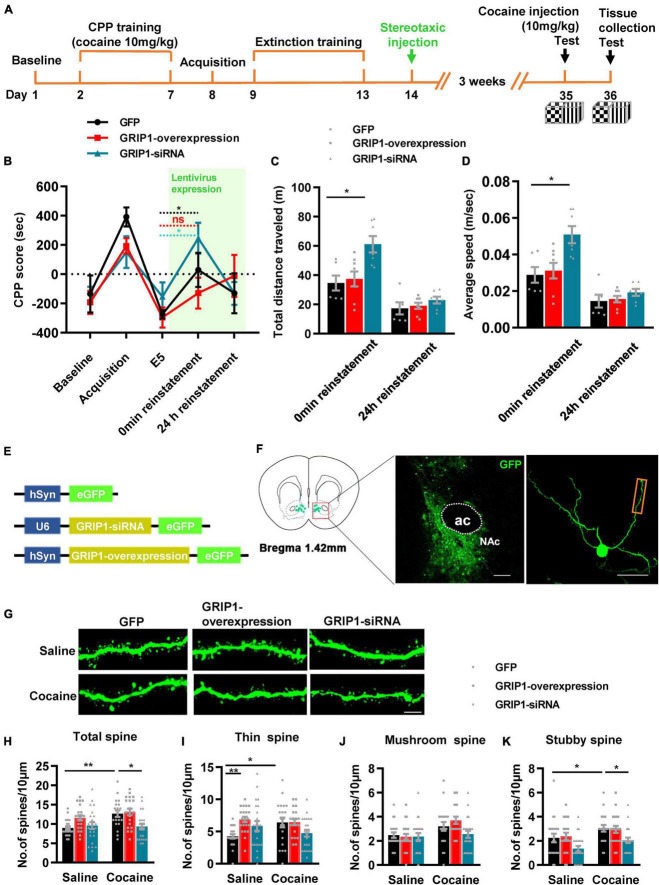
GRIP1 interference enhances cocaine reinstatement but blocks associated spine plasticity. **(A)** Experimental design for the behavioral tests and spine analysis. Two-compartment unbiased CPP models were used. **(B)** GRIP1-siRNA enhanced reinstatement to cocaine place preference, while the GRIP1-overexpression group and the GFP control did not exhibit cocaine reinstatement in the unbiased CPP test. **(C,D)** Total distance traveled and average speed during 0 min reinstatement. **(E)** Viral vectors used to interfere with the expression of GRIP1. **(F)** Representative images of the injection site (left) and spine densities on terminal dendrites (right). **(G)** Representative images of GFP-labeled dendrites. **(H–K)** The statistical data for stubby spine, mushroom spine, thin spine, and total spine density. *n* = 20–30 dendrites obtained from 8–10 mice per group. *P < 0.05, **P < 0.01, and ***P < 0.001.

To verify our hypothesis, we injected GRIP1-siRNA and GRIP1-overexpressing lentivirus into the NAc after cocaine extinction; after 3 weeks of recovery, one 10 mg/kg cocaine injection was used to induce reinstatement (Figures 6A,E). The behavioral test results showed that GRIP1-siRNA exhibited cocaine reinstatement (Figure 6B, *t* = 2.293, *P* = 0.045) and locomotor activity (Figure 6C, *F*_(2,18)_ = 7.397, *P* = 0.005; Figure 6D, *F*_(2,18)_ = 7.396, *P* = 0.005) at 0 min reinstatement on Day 35 compared to the GFP control group, while GRIP1-overexpressing mice did not exhibit cocaine reinstatement behavior (Figures 6B–D, all *P*’s > 0.05). The spine analysis results showed that GRIP1-siRNA blocked the cocaine-induced increase in total spine density of MSNs in NAc at 24 h reinstatement on Day 36 (Figures 6G,H, two-way ANOVA followed by simple main effects analysis, *F*_drug(1,121)_ = 7.1, *P* = 0.009; *F*virus_(2,121)_ = 7.308, *P* = 0.001; *F*_interaction(2,121)_ = 3.397, *P* = 0.037); the blockage by GRIP1-siRNA was driven by a decrease in thin spine (Figures 6F,G,I, two-way ANOVA followed by simple main effects analysis, *F*_drug(1,121)_ = 0.189, *P* = 0.665; *F*virus_(2,121)_ = 3.587, *P* = 0.031; *F*_interaction(2,121)_ = 4.529, *P* = 0.013), mushroom spine (Figures 6G,J, two-way ANOVA, *F*_drug(1,121)_ = 10.332, *P* = 0.002; *F*_virus(2,121)_ = 2.063, *P* = 0.132; *F*_interaction(2,121)_ = 2.054, *P* = 0.133), and stubby spine (Figures 6G,K, two-way ANOVA, *F*_drug(1,121)_ = 9.16, *P* = 0.003; *F*_virus(2,121)_ = 8.034, *P* = 0.001; *F*_interaction(2,121)_ = 0.091, *P* = 0.913) density. Our data indicated that GRIP1 in the NAc regulated cocaine reinstatement and associated spine plasticity.

### Glutamate receptor-interacting protein 1-GluA2 in D1-medium spiny neurons and D2-medium spiny neurons of the nucleus accumbens differentially regulates cocaine reinstatement and associated spine plasticity

**FIGURE 7:**
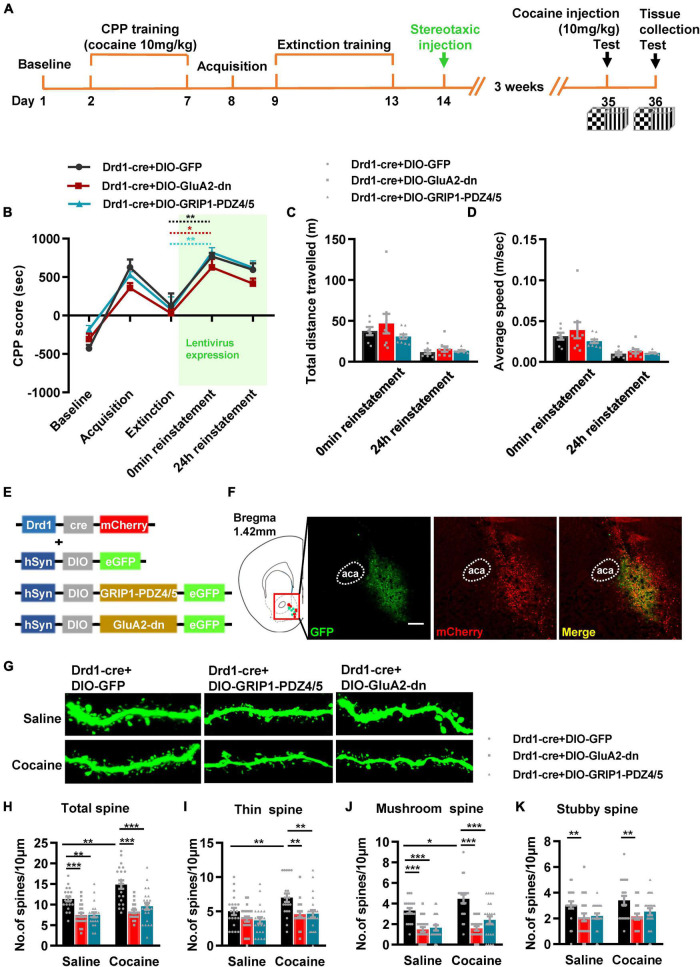
Interference with GRIP1 and GluA2 interaction in D1-MSNs does not affect cocaine-induced reinstatement but blocks the associated spine plasticity of D1-MSNs. **(A)** Experimental design for the behavioral tests and spine analysis. Two-compartment unbiased CPP models were used. **(B)** GluA2-dn and GRIP1-PDZ4/5 expressed in D1-MSNs did not affect cocaine-induced reinstatement in the unbiased CPP test. **(C,D)** Total distance traveled and average speed during 0 min reinstatement. **(E)** Viral vectors used to interfere with the interaction of GRIP1 and GluA2 in D1-MSNs. **(F)** Representative images of the injection site. **(G)** Representative images of GFP-labeled dendrites. **(H–K)** The statistical data for stubby spine, mushroom spine, thin spine, and total spine density. *n* = 20–30 dendrites obtained from 8–10 mice per group. *P < 0.05, **P < 0.01, and ***P < 0.001.

Next, we evaluated the role of GRIP1 in D1-MSNs and D2-MSNs in regulating cocaine reinstatement. Drd1-Cre AAV and GRIP1-PDZ4/5 or GluA2-dn lentivirus cocktails (Figures 7E,F) were injected into the NAc after extinction (Figure 7A). After 3 weeks of recovery, a 10 mg/kg cocaine injection was used to induce reinstatement. The behavioral results showed that both GluA2-dn and GRIP1-PDZ4/5 in D1-MSNs had similar cocaine-induced reinstatement (Figure 7B, *F* = 1.756, *P* = 0.193) and locomotor activity (Figure 7C, *F*_(2,23)_ = 1.147, *P* = 0.335; Figure 7D, *F*_(2,23)_ = 1.204, *P* = 0.318) at 0 min and 24 h reinstatement compared to the GFP control group. Meanwhile, the spine analysis results showed that both GRIP1-PDZ4/5 and GluA2-dn blocked the cocaine-induced increase in total spine density of D1-MSNs (Figures 7G,H, two-way ANOVA, *F*_drug(1,123)_ = 14.191, *P* < 0.001; *F*_virus(2,123)_ = 32.447, *P* < 0.001; *F*_interaction(2,123)_ = 1.773, *P* = 0.174); this blockage was driven by a decrease in thin spine (Figures 7G,I, two-way ANOVA, *F*_drug(1,123)_ = 7.475, *P* = 0.007; *F*_virus(2,123)_ = 33.367, *P* < 0.001; *F*_interaction(2,123)_ = 1.58, *P* = 0.21), mushroom spine (Figures 7G,J, two-way ANOVA, *F*_drug(1,123)_ = 9.288, *P* = 0.003; *F*_virus(2,123)_ = 8.688, *P* < 0.001; *F*_interaction(2,123)_ = 0.937, *P* = 0.395), and stubby spine (Figures 7G,K, two-way ANOVA, *F*_drug(1,123)_ = 1.191, *P* = 0.277; *F*_virus(2,123)_ = 8.482, *P* < 0.001; *F*_interaction(2,123)_ = 1.81, *P* = 0.835) density in D1-MSNs. Our data indicate that the interference of the interaction between GRIP1 and GluA2 in D1-MSNs blocked cocaine reinstatement-associated spine plasticity but did not strongly promote cocaine reinstatement behavior.

**FIGURE 8:**
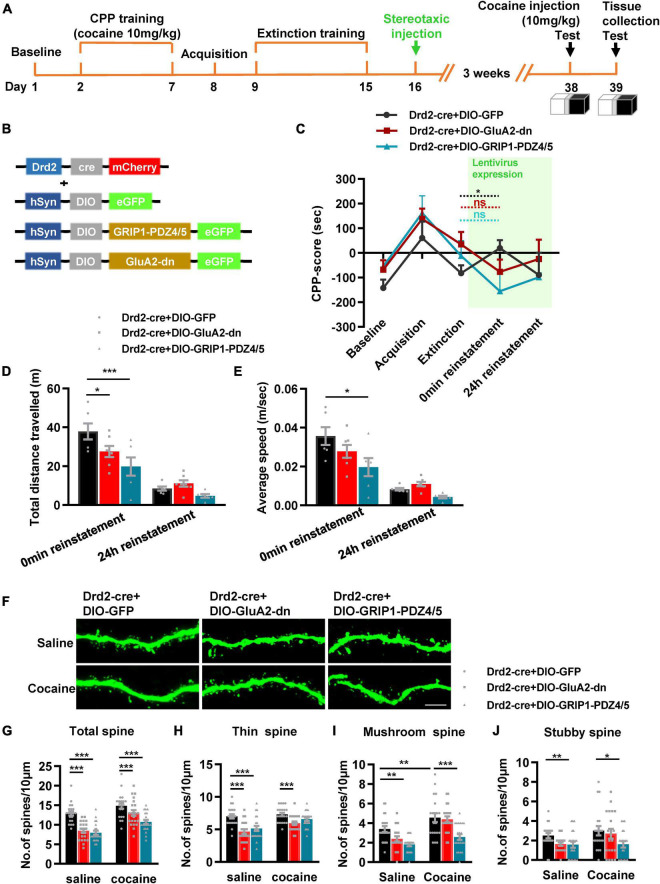
Interference of GRIP1 and GluA2 interaction in D2-MSNs blocks cocaine-induced reinstatement and deceases spine density of D2-MSNs. **(A)** Experimental design for the behavioral tests and spine analysis. Three-compartment biased CPP models were used. **(B)** Viral vectors used to interfere with the interaction of GRIP1 and GluA2 in D2-MSNs. **(C)** GluA2-dn and GRIP1-PDZ4/5 expressed in D2-MSNs blocked cocaine-induced reinstatement. **(D,E)** Total distance traveled and average speed during 0 min reinstatement. **(F)** Representative images of GFP-labeled dendrites. **(G–J)** The statistical data for stubby spine, mushroom spine, thin spine, and total spine density. *n* = 20–30 dendrites obtained from 8–10 mice per group. *P < 0.05, **P < 0.01, and ***P < 0.001.

Next, we used Drd2-cre AAV to initiate GRIP1-PDZ4/5 or GluA2-dn in D2-MSNs following the procedure in Figures 8A,B. The behavioral test results showed that both GluA2-dn and GRIP1-PDZ4/5 in D2-MSNs blocked cocaine reinstatement (Figure 8C, reinstatement vs. extinction, cocaine + Drd2-Cre + DIO-GluA2-dn: *t* = 1.422, *P* = 0.193; cocaine + Drd2-Cre + DIO-PDZ4/5: *t* = 0.166, *P* = 0.296; but cocaine + Drd2-Cre + DIO-GFP: *t* = 3.558, *P* = 0.016) and locomotor activity (Figure 8D, *F*_(2,16)_ = 5.142, *P* = 0.019; Figure 8E, *F*_(2,16)_ = 3.531, *P* = 0.054). Meanwhile, the spine analysis results showed that both GRIP1-PDZ4/5 and GluA2-dn decreased the total spine density of D2-MSNs in both baseline and cocaine circumstances (Figures 8F,G, two-way ANOVA, *F*_drug(1,119)_ = 12.793, *P* = 0.001; *F*virus_(2,119)_ = 28.164, *P* < 0.001; *F*_interaction(2,119)_ = 0.486, *P* = 0.617; Bonferroni test, cocaine + Drd2-Cre + DIO-GluA2-dn vs. cocaine + Drd2-Cre + DIO-GFP, *P* < 0.001; Cocaine + Drd2-Cre + DIO-GRIP1-PDZ4/5 vs. cocaine + Drd2-Cre + DIO-GFP, *P* < 0.001), but GRIP1-PDZ4/5 decreased stubby spine density (Figures 8F,J, cocaine + Drd2-Cre + DIO-PDZ4/5 vs. cocaine + Drd2-Cre + DIO-GFP, *P* = 0.01), and mushroom spine density (Figures 8F,I, cocaine + Drd2-Cre + DIO-PDZ4/5 vs. cocaine + Drd2-Cre + DIO-GFP, *P* < 0.001) of D2-MSNs; GluA2-dn decreased thin spine density (Figures 8F,H, *F*_drug(1,119)_ = 5.364, *P* < 0.001; *F*_virus(2,119)_ = 17.943, *P* < 0.001; *F*_interaction(2,119)_ = 1.874, *P* = 0.158; cocaine + Drd2-Cre + DIO-GluA1-dn vs. Cocaine + Drd2-Cre + DIO-GFP, *P* = 0.009) of D2-MSNs. Our data indicated that the interference of the interaction between GRIP1 and GluA2 in D2-MSNs reduced cocaine reinstatement and spine density; it played an opposite regulatory role to that in D1-MSNs.

## Discussion

**FIGURE 9:**
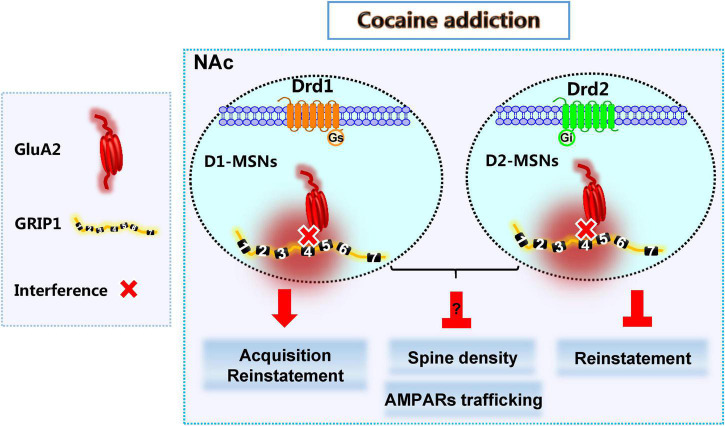
GRIP1 in D1-MSNs and D2-MSNs differentially regulates cocaine acquisition, reinstatement, and associated spine plasticity.

Here, we show that decreased expression of GRIP1 by GRIP1-siRNA in the NAc potentiated cocaine-seeking behavior in acquisition, which is mainly driven by the interference of GRIP1 and GluA2 interaction in D1-MSNs, and sustained cocaine-seeking behavior in reinstatement, which can be blocked by the interference of GRIP1 and GluA2 interaction in D2-MSNs. Decreased GRIP1 expression due to GRIP1-siRNA in the NAc decreased basal spine density and thereby influenced cocaine-induced increase in spine density. When there was interference of the GRIP1-GluA2 interaction specifically in D1- and D2-MSNs, spine density still stayed on a low level in cocaine acquisition due to decreased basal spine density; or directly blocked cocaine-induced increase in spine density in reinstatement (Figure 9). These data demonstrate that cell-type-specific GRIP1 contributes to the encoding of cocaine acquisition and reinstatement, and decreased GRIP1 in D1-MSNs plays a leading role in mediating cocaine acquisition and reinstatement.

D1-MSNs and D2-MSNs in the NAc usually play different regulatory roles in drug addiction (Hikida et al., 2010; James et al., 2013; Chandra et al., 2015; Khibnik et al., 2016; Barrientos et al., 2018). Our results show that the interaction of GRIP1 and GluA2 in D1-MSNs and D2-MSNs differentially regulated cocaine acquisition and reinstatement (Figures 3, 4, 7, 8), which further proves the different roles of D1-MSNs and D2-MSNs in drug addiction; focusing on cell subclasses is necessary to study the role of proteins in diseases. Meanwhile, our behavioral data indicated that interference of the GRIP1-GluA2 interaction in D1-MSNs has a similar tendency to the results obtained when all types of neuronal targets are interfered by GRIP1-siRNA (Figures 2, 3, 6, 7), which illustrates that GRIP1 in D1-MSNs plays a leading role in cocaine addiction. Furthermore, D2-MSNs seem to be blunted by low-dose or low-intensity drug stimulation (Madayag et al., 2019). In the CPP model, which uses a 20 mg/kg cocaine injection every other day to detect cocaine acquisition, cocaine-induced spine plasticity mainly occurs in D1-MSNs and not D2-MSNs (Figures 3, 4). When we use a 10 mg/kg cocaine regimen to construct the cocaine reinstatement model, the distal dendritic spines in D1-MSNs also show greater plasticity in D1-MSNs than in D2-MSNs, showing that the total, thin, and mushroom spine density increase in D1-MSNs after cocaine treatment; only mushroom spine density increases, with no change in total and thin spine density in D2-MSNs after cocaine reinstatement regimens (Figures 7, 8).

GRIP1 in neurons is required for long-term depression (LTD) expression in the cerebellum (Takamiya et al., 2008) and hippocampus (Dickinson et al., 2009); LTD is very important in promoting forgetting (Nabavi et al., 2014; Awasthi et al., 2019). Combined with our findings that GRIP1 knockdown by GRIP1-siRNA enhanced cocaine acquisition and reinstatement (Figures 2, 6), which is consistent with findings of previous studies using GRIP1 conditional knockout mice (Briand et al., 2014). We speculate that loss of GRIP1 expression may cause deficits in forgetting cocaine-associated memories, thus maintaining cocaine acquisition and reinstatement at a high level. Meanwhile, it has been reported that LTD is associated with phosphorylation of Ser880 within the GluR2 C-terminal PDZ ligand, which decreases GluR2 binding to GRIP1 (Chung et al., 2000; Kim et al., 2001). In our research, we found that interfering with the interaction between GluA2 and GRIP1 by GluA2-dn or GRIP1-PDZ4/5 in D1-MSNs enhanced cocaine acquisition (Figure 3) and did not change the tendency toward cocaine reinstatement (Figure 7). This further confirms that the blockage of the interaction between GluA2 and GRIP1 may cause a deficit in AMPA receptor internalization during LTD, leading to a deficit in forgetting cocaine-associated memories. Meanwhile, we found that GRIP1 siRNA and overexpression had the same effect on behavior (Figures 2B, 3B), which further proves our conclusion in previous research that GRIP1 imbalance, both overexpression and blockade, can drive AMPA receptor endocytosis and exocytosis bidirectionally (Wang et al., 2022), therefore probably inducing a similar effect on behavior.

GRIP1 is abundantly expressed in both glutamatergic and GABAergic synapses (Li et al., 2005), suggesting a role in regulating both excitatory and inhibitory synaptic functions. In the present research, we found that GRIP1 interference by GRIP1-siRNA and GRIP1 overexpression differentially regulated spine plasticity in cocaine acquisition and reinstatement. Different cocaine doses and regimens may have different degrees of influence on glutamatergic and GABAergic synapses, which may lead to differences in spine plasticity. Moreover, this illustrates that morphological changes and behavioral performance are independent phenomena in cocaine addiction.

Spine shape may have an association with learning and memory; among these, thin spines are highly variable and respond to changes in circumstance or drug addiction and are considered to be “learning spines,” while stubby or mushroom spines are relatively stable and considered to be “memory spines” (Bourne and Harris, 2007). We found that interference with GRIP1 mainly influences thin spine plasticity in cocaine acquisition (Figures 2–4) and stubby and mushroom spine plasticity in cocaine reinstatement (Figures 6–8). Both affect cocaine-induced CPP behavior; for example, interference in the interaction in D1-MSNs results in positive regulation, while interference in D2-MSNs results in negative regulation. Accordingly, cocaine acquisition training could mainly be a learning process, while reinstatement training could be a memory process. Thus, GRIP1 is involved in both cocaine-associated learning and memory, consistent with the finding that GRIP1 regulates electric shock-related learning and memory in the hippocampus (Tan et al., 2020). Meanwhile, the circuit from the hippocampal CA1 to the NAc mediates cocaine-associated memory (Zhou et al., 2019, 2020); hence, GRIP1 in the NAc may be involved in this circuit to adjust learning and memory in cocaine-induced CPP behavior.

Loss of GRIP1 expression in neurons leads to decelerated recycling of GluA2 to the synaptic membrane in hippocampal neurons (Mao et al., 2010). Furthermore, cocaine acquisition-induced formation of thin spines is mainly found in silent synapses that lack AMPARs (Matsuzaki et al., 2001; Huang et al., 2009), while cocaine reinstatement can be mediated by GluA2-lacking Ca^2+^ permeable AMPARs (CP-AMPARs) that lack GluA2 subunits (Lee et al., 2013). Our results show that GRIP1 expression decreases in both cocaine acquisition and reinstatement (Figures 1, 5) that may cause deficits in GluA2 trafficking in neurons and thus affect AMPARs lacking thin spine plasticity in cocaine acquisition and GluA2-lacking CP-AMPARs insertion in the postsynaptic membrane, which induce cocaine reinstatement. GRIP1 deficits in cocaine acquisition and reinstatement may partly explain why cocaine acquisition mainly induces thin spine plasticity and why CP-AMPARs are inserted into the postsynaptic membrane during cocaine reinstatement. Combined with our results that GRIP1 has not always played a significant role on spine plasticity in cocaine acquisition, we speculated that the effect of GRIP1 on cocaine acquisition behavior may be either through influencing the basal spine density or by regulating AMPA receptors trafficking in neurons without affecting the spine plasticity in cocaine acquisition. Meanwhile, in cocaine reinstatement, although GRIP1 regulated cocaine-induced spine plasticity, AMPA receptors trafficking may also involve in cocaine reinstatement.

In Figure 6, the reinstatement CPP score are not obviously high, this may be caused by several reasons. First, the CPP reinstatement is dose and time point dependent (Bobzean et al., 2010; Kennedy, 2016). We have used a relatively low dose (10 mg/kg), and the extinction of mice on extinction Day 5 is relatively complete and thorough, and the CPP score has decreased to a negative value. Therefore, the most ideal level of reinstatement has not been achieved when primed by low-dose 10mg/kg cocaine. Secondly, it is possible that during extinction the mice developed avoidance to the cocaine-paired side and the reinstatement test shows that mice overcame the avoidance, if this is the reason, maybe GRIP1-siRNA is not affecting reward learning but the will to “pay a price” in order to receive cocaine. These hypothesis need our further confirmation.

Above all, our results show that GRIP1 in the NAc regulates cocaine acquisition and reinstatement, which is led by the interaction between GRIP1 and GluA2 in D1-MSNs. Meanwhile, GRIP1 and GluA2 interactions in D1- and D2-MSNs play different roles in cocaine acquisition and reinstatement. GRIP1 may be a drug development target for the treatment of addiction and relapse. Further studies are required to explain why GRIP1 deficits play different roles in cocaine-induced AMPAR trafficking in acquisition and reinstatement.

## Data availability statement

The raw data supporting the conclusions of this article will be made available by the authors, without undue reservation.

## Ethics statement

The animal study was reviewed and approved by Institutional Animal Care and Use Committee of the Southern Medical University.

## Author contributions

HC and LC: conceptualization, methodology, formal analysis, investigation, data curation, visualization, and writing—original draft. ZY, JY, YL, YX, and JW: conceptualization, methodology, data analysis, and writing—original draft. LZ: conceptualization, methodology, resources, writing—review and editing. JL and GW: conceptualization, methodology, supervision, resources, writing—original draft and review and editing, and funding acquisition. All authors contributed to the article and approved the submitted version.
